# A new minimally invasive method for anatomic reconstruction of the lateral ankle ligaments with a Tightrope system

**DOI:** 10.1007/s00402-018-2955-4

**Published:** 2018-06-06

**Authors:** Yongxing Cao, Yang Xu, Yuan Hong, Xiangyang Xu

**Affiliations:** Department of Orthopedics, Shanghai Ruijin Hospital North, Shanghai Jiao Tong University School of Medicine, No. 999, Xiwang Rd, Shanghai, 201800 China

**Keywords:** Allograft, Anatomy, Ankle, Ligament, Minimal invasive, Tightrope

## Abstract

**Background:**

Several minimally invasive anatomic reconstruction techniques of the lateral ligaments have been introduced for the treatment of chronic lateral ankle instability. However, these strategies may not always follow accurate ligament anatomic attachments, especially in the construction of the fibular bone tunnels.

**Objectives:**

This study reported a new percutaneous technique for reconstruction of the ligaments of lateral ankle anatomically with a Tightrope system.

**Methods:**

From April 2016 to August 2016, 25 ankles of 24 patients with chronic ankle instability underwent our new percutaneous anatomic reconstruction of the lateral ligaments with a Tightrope system. The operation was performed through several small incisions. The fibular tunnel was made obliquely from the anteromedial side of lateral malleolus tip towards retro-malleolar cortex. The graft was fixed in the tunnel with the help of a Tightrope system. The calcaneal tunnel and talar tunnel were made as our previous method. The mean final follow-up was 12.2 months (range 10–14). Visual Analogue Scale for pain, American Orthopaedic Foot and Ankle Society score, and patients’ subjective satisfaction were used to measure clinical outcomes. Preoperative and postoperative stress tests were performed and radiographic parameters were measured.

**Results:**

The Visual Analogue Scale decreased from 3.0 ± 1.4 to 1.3 ± 0.8 at the last follow-up (*p* < 0.01). The American Orthopaedic Foot and Ankle Society score was improved from 70.2 ± 5.4 preoperatively to 92.4 ± 5.3 at the final follow-up (*p* < 0.01). Radiologically, the mean anterior talar displacement was 13.1 ± 2.7 mm preoperatively versus 5.6 ± 1.3 mm at last follow-up (*p* < 0.01),and the mean varus talar tilt angle was 15.0° ± 2.4° preoperatively versus 5.6° ± 1.9° at the last follow-up (*p* < 0.01). Patients were satisfied (‘excellent’ or ‘good’) in 23 ankles (92%). Two patients reported residual instability but less apprehension than the preoperative condition.

**Conclusions:**

Percutaneous anatomic reconstruction of the lateral ligaments of the ankle with a Tightrope system is an anatomic and effective procedure for the treatment of chronic lateral ankle instability.

## Introduction

Chronic ankle instability (CAI) is one of the most common problems in foot and ankle surgery. Despite adequate primary treatment including immobilization and physical therapy, approximately 20–40% of patients present with persistent instability and require surgical intervention. Among the techniques used, anatomic reconstruction is one of the most commonly reported, with good to excellent results. However, these strategies may not always follow accurate ligament anatomic attachments, especially in the construction of the fibular bone tunnels. After some years of experience with open approaches and percutaneous techniques, we adopted a new minimally invasive technique for reconstruction of the ligaments of lateral ankle anatomically with a Tightrope system. The rationale for this technical stratagem is based on cadaver studies that demonstrated the original footprints of anterior talofibular ligament (ATFL) and calcaneofibular ligament (CFL) on the lateral malleolus [1, 2]. The subjective and objective function as well as radiographic changes of the ankle with this minimally invasive procedure was evaluated in this study.

## Methods

We analyzed 25 ankles of 24 patients who underwent percutaneous lateral ligaments reconstructions using allograft with a Tightrope system between April 2016 and August 2016 for the treatment of CAI. These patients had ankle instability or repetitive ankle sprain injuries despite a minimum of 6 months of non-operative treatment with a rehabilitation program focused on proprioceptive training and peroneal strengthening.

### Indications for ligament reconstruction

All patients included in this study satisfied at least two of the following criteria: (1) generalized ligamentous laxity, (2) previously failed reconstruction of the lateral ligaments, (3) obesity (body mass index more than 25), (4) high demand heavy athletes or laborers, (5) poor quality tissue during the intraoperative evaluation, (6) severe ankle instability, significant ankle laxity with a ≥ 10° difference in talar tilt angle when compared with the opposite side or an absolute talar tilt angle ≥ 15°, and more than 10 mm of anterior talar displacement. All patients with the following surgery contraindications were excluded: (1) ankle infection, (2) fracture, (3) ankle arthritis > grade 2 according to Morrey and Wiedeman classification, (4) functional instability without mechanical instability on stress radiographs.

### Operative technique

After general or continual epidural anesthesia, the patients were supine with a pneumatic tourniquet on the proximal thigh. A pad was routinely placed under the affected buttock to rotate the limb medially.

We performed an arthroscopic examination of the ankle immediately before reconstruction of the lateral ligaments to evaluate and treat any accompanying intraarticular lesions through the standard antero-medial and antero-lateral arthroscopy portals. The ankle joint was explored thoroughly to identify all lesions (condition of the anterior talofibular ligament, medial collateral ligament, synovium, cartilage, and bone).

**Fig. 1:**
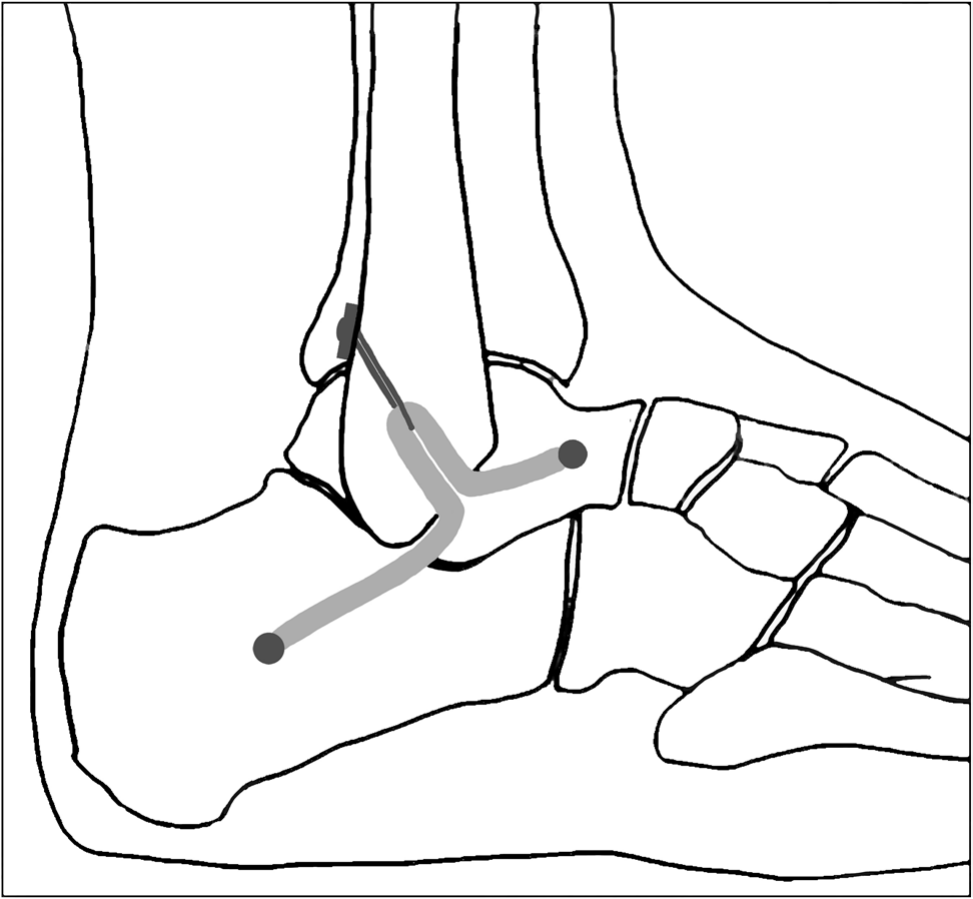
Schematic drawing of the reconstruction method

Figure 1 shows sagittal illustrations of the reconstruction method performed in the lateral positon.

**Fig. 2:**
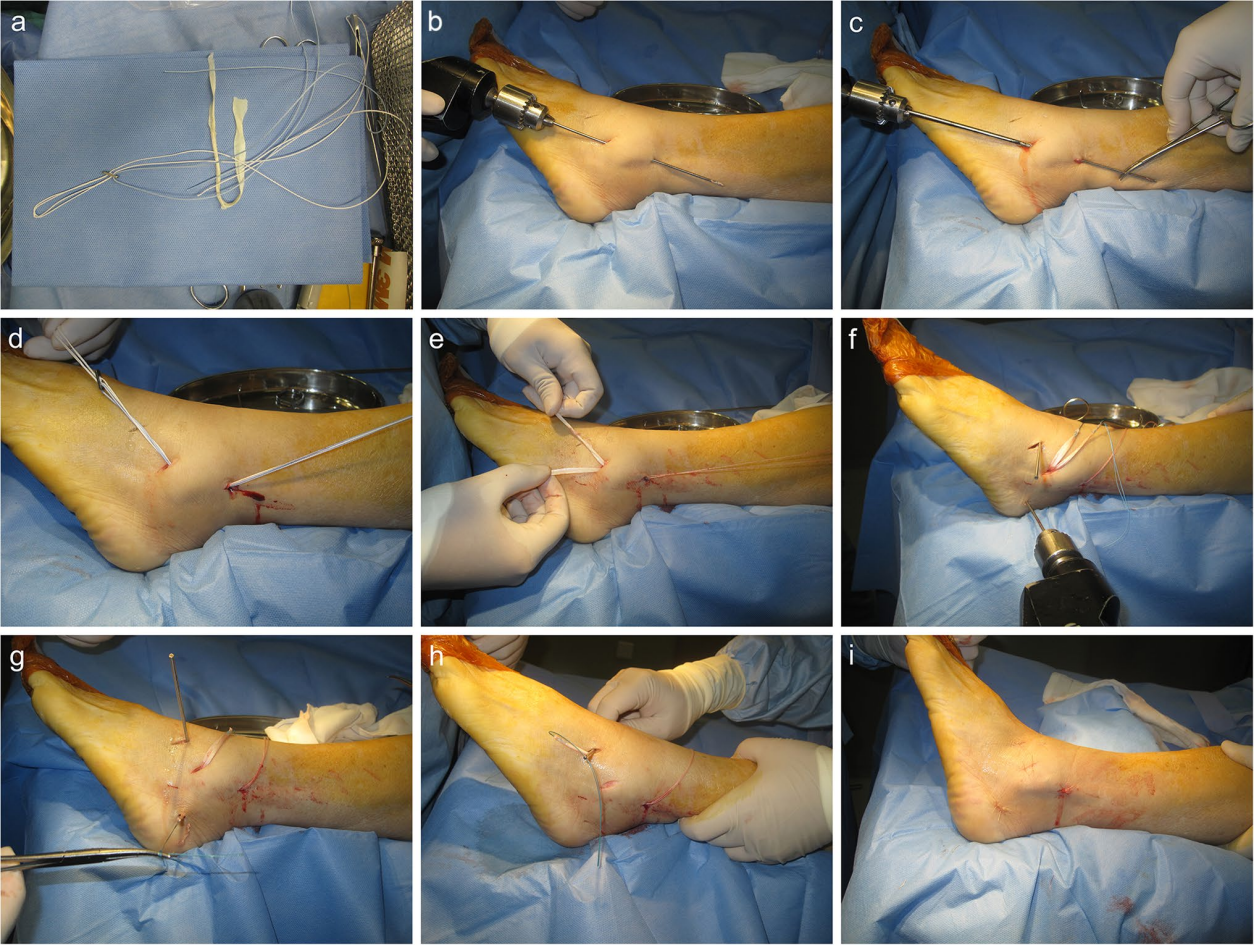
Percutaneous anatomic reconstruction of the lateral ligaments of the ankle with a Tightrope system. **a** Semitendinosus allograft ligament and Tightrope system. **b, c** Creating the fibular tunnel. **d, e** Introduction of allograft and positioning of the cortical button. **f** Creating the talar and calcaneal tunnels. **g, h** Passing and fixation the graft ends to the talar and calcaneal tunnels. **i** Sutured incisions

The semitendinosus allograft ligament (Osteorad Ltd, Shanxi, China) and Tightrope system (ACL TightRope^®^ RT, Arthrex, USA) was used for anatomic reconstruction of the anterior talofibular ligament and calcaneofibular ligament using a percutaneous minimally invasive technique (Fig. 2a).

At the recipient site of the ankle, we made three small incisions of 5 mm each at the anteromedial side of the lateral malleolus tip, talar neck, and the middle portion of the calcaneus.

A guide wire was introduced through the center of medial and lateral cortex of lateral malleolus in an upwards and posterior direction from the anteromedial side of lateral malleolus tip, towards to retro-malleolar cortex about 2.5 cm above the lateral malleolus tip (Fig. 2b). To ensure proper positioning of the cortical button, a fourth incision was made at the penetrating site of the guide wire tip, just behind the posterior fibular cortex. A fibular tunnel 4.5 mm in diameter was created over the guide wire using a drill bit while protecting of the peroneal tendons (Fig. 2c). It is critical to ensure that the tunnel was placed in the center of the fibula in the coronal plane to minimize the risk of tunnel compromise or blowout.

The allograft was trimmed to a minimum of 14 cm (in length) × 8 mm (in width) × 1 mm (in thickness) after cryogenic processing. Load the graft through the implants by folding it symmetrically over the Tightrope loops. Stitch approximately 1.5 cm of each graft end with a high-strength nonabsorbable no. 2 suture.

The cortical button leading line was passed through the fibular tunnel with the help of a guide eyelet wire (Fig. 2d). Then the cortical button was advanced out of the fibular tunnel. The graft was then pulled back to confirm the button was seated. After that, the cortical button was tightened in its position (Fig. 2e).

For positioning of calcaneal tunnel, an eyelet wire was introduced towards the posterior, inferior, and medial edge of the calcaneus as described by Xu et al. [3]. A 4.5 mm drill was used to create a tunnel that was then widened to 7 mm with an approximate depth of 2.5 cm. The talar tunnel was made by a similar method (Fig. 2f). The ends of the tendon were then passed above the bone surface to the incisions at the talar neck and calcaneus (Fig. 2g, h).

**Fig. 3:**
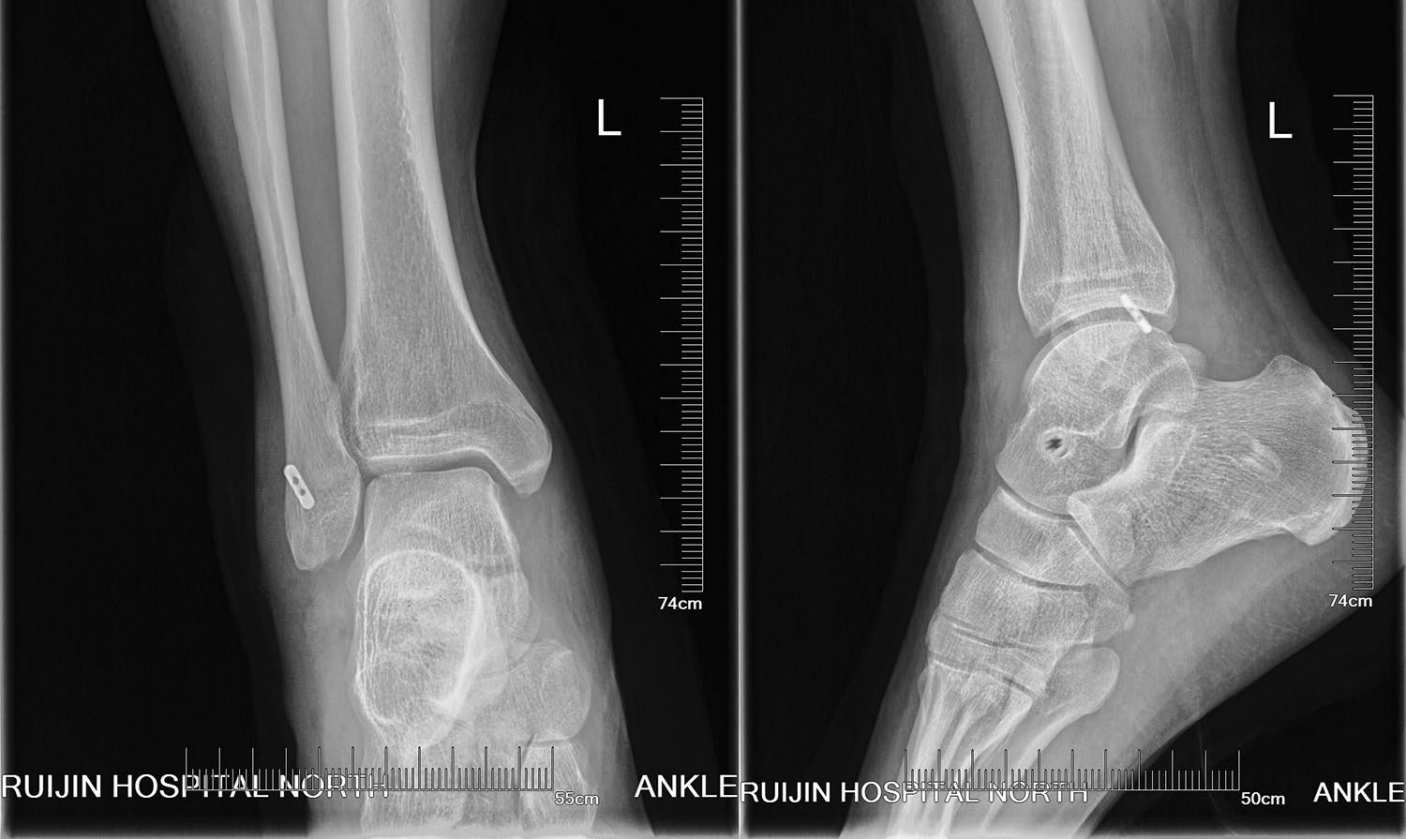
Postoperative radiographic images showing the locations of the cortical button and interference screws

With the ankle and foot in the neutral position, two 7 mm × 23 mm biodegradable inference screws (BioCryl^®^, Depuy Miteck, Raynham, MA) were used to fix each end of the tendon graft in the talar neck and calcaneus in a lateral-to-medial direction along the guide wire while maintaining tension on the graft (Fig. 2i). The locations of cortical button and interference screws were examined under fluoroscopy (Fig. 3).

### Rehabilitation protocol

Postoperatively, the affected ankle was immobilized in a valgus position and a weight free manner using a U-shaped short-leg cast. Isometric dorsiflexion strengthening of the ankle was allowed to reduce stiffness at 3 days after operation. The cast was changed to an ankle orthosis (VACO cast, Company OPED, Germany) at 2 weeks after surgery. The patient was advanced to partial weight bearing by 3 weeks. Full weight-bearing started at 6 weeks postoperatively. The ankle orthosis was removed at 10 weeks after operation, and the patients transitioned to a normal shoe with a soft brace. Jogging was resumed at 12 weeks after operation.

### Clinical evaluation

All patients were evaluated from both the clinical and radiographic perspective. The clinical outcomes were evaluated with Visual Analogue Scale (VAS) for pain, American Orthopaedic Foot and Ankle Society-Ankle and Hindfoot (AOFAS-AH) score, and patients’ subjective satisfaction. The questionnaires of VAS and AOFAS-AH were completed before surgery and at last follow-up. Patients’ subjective satisfaction level was graded as excellent, good, fair, or poor. Excellent is referred to full activity, including strenuous sports, with no pain, swelling, or giving way of the ankle. Good is referred to occasional aching of the ankle but only after strenuous exercise, no giving way or feeling of apprehension. Fair is referred to residual instability and remaining apprehension but less instability and apprehension as compared with the patient’s ankle condition before surgery. Poor is referred to recurrent ankle instability and giving way, unchanged or worse in normal activities with episodes of pain and swelling. Preoperative and postoperative stress radiographs were taken using a TELOS stress device. Radiographic parameters included the anterior talar displacement and varus talar tilt angle between bony surfaces of the talus and tibia.

### Statistical methods

Statistical analysis was performed using SPSS software version 18.0 (SPSS Inc., Chicago). Changes in the VAS, AOFAS-AH score, and radiographic parameters before and after operation were analyzed by Wilcoxon test. *P* values less than 0.05 were considered statistically significant.

## Results

This study group included 9 males and 15 females. The average age at the operation time was 30.8 years (range 18–50). The mean final follow-up was 13.9 months (range 12–16). The VAS decreased from 3.0 ± 1.4 before surgery to 1.3 ± 0.8 at the last follow-up (*p* < 0.01). The AOFAS-AH score was significantly improved from 70.2 ± 5.4 preoperatively to 92.4 ± 5.3 at the final follow-up (*p* < 0.01). Radiologically, the mean anterior talar displacement was 13.1 ± 2.7 mm preoperatively versus 5.6 ± 1.3 mm at last follow-up (*p* < 0.01), and the mean varus talar tilt angle was 15.0° ± 2.4° preoperatively versus 5.6° ± 1.9° at the last follow-up (*p* < 0.01). Patients were satisfied (‘excellent’ or ‘good’) in 23 ankles (92%). The patient satisfaction level for the other two cases was ‘fair’. They reported residual instability but less apprehension than the preoperative condition.

Surgical complications were reported in three patients. No superficial wound infections occurred. One patient had injury of the branch of superficial peroneal nerve and a sensory disturbance on the lateral aspect of the foot and did not influence the final clinical result. Another two patients reported soft tissue irritation from the cortical button. No granuloma formation or osteolysis in adjacent bone occurred in the patients. The satisfaction in one of them was fair, because of chronic ankle pain and residual instability. There has not been a need for reoperation in any of the patients.

## Discussion

In this study, the patients achieved satisfactory clinical results after lateral ankle reconstruction using a percutaneous anatomic reconstruction technique with a Tightrope system. Our reconstruction method restored the normal anatomy by positioning the allograft at the original point ligament origin and insertion. There are two patients reported a residual instability on uneven ground, but they thought it was better than the preoperative condition. This study supports the effectiveness of this approach in this group of patients with severe instability.

To date, many surgical techniques have been described to manage CAI. These techniques and their modifications fall into three categories: non-anatomic reconstruction, anatomic repairment, and anatomic reconstruction. Non-anatomic reconstruction uses various configurations of local tendon grafts to accomplish the restriction function of the ligament without repair of the injured ligaments. Several techniques have been described, including partial or complete tenodesis from the peroneal tendon or Achilles tendon; or allografts mimicking the function of the original ligament such as the Chrisman–Snook (CS) [4], the Evans procedure [5] and the Watson Jones procedure [6]. Anatomic repairment is to restore normal anatomy and joint mechanics by in situ repair of the injured ligament. Anatomic repairment includes repair ligaments by either shortening and reattaching them to the bony surfaces, or augmenting them with surrounding structures to enhance the repairment. A good example is the classic Brostrom–Gould procedure [7], which empowers the original ligaments with the extensor retinaculum and has proved to be a strong procedure without sacrificing other anatomic structures. Anatomic reconstruction procedures use tendon grafts to recreate joint biomechanics anatomically by replicating the anatomic positions of the ATFL and CFL origin and insertion sites. They vary in the means by which they attain that positioning, including the number and angle of tunnels in the fibula and the fixation techniques selected in each bone tunnel location.

Non-anatomic techniques have been used in the past, but currently are not the procedure of choice, as such procedures do not reestablish the ankle kinematics, but stabilize the ankle and results in ankle stiffness [8, 9]. Now Brostrom–Gould procedure is considered to be the gold standard for surgical treatment of CAI [7, 10–13]. However, anatomic repairment does not fully address special conditions such as severe instability or revision surgery. This procedure may not provide adequate stability and lead to recurrence using the weakened and scarred remnants. Subsequently, researchers have described several anatomic reconstruction procedures using autograft or allograft tendon [14, 15]. Studies have demonstrated that anatomic reconstruction can improve lateral ankle instability and restore normal ankle motion [16–19]. Besides, there has been a recent trend of minimally invasive anatomic reconstruction of the lateral ankle ligaments for CAI, which has been found both feasible and reproducible. However, there is still large room to improve this technically demanding procedure.

Some researchers, including our team, have reported a few minimally invasive techniques to reconstruct lateral ankle ligament. However, these strategies may not always follow accurate ligament anatomic attachments, especially in the construction of the fibular bone tunnels. Panchbhavi [19], Kim et al. [20] and Youn et al. [21] made a straight fibular tunnel in an anterior to posterior or otherwise direction, while Xu and Wang et al. [3, 22] made a ‘Γ’ shaped fibular tunnel.

To perform an anatomic reconstruction, the anatomy must be well understood. When performing an anatomic reconstruction of the lateral ligament complex, the surgeon has little guidance on where to place bony tunnels. Based on the research of eight unpaired fresh-frozen cadaver feet, Neuschwander et al. [2] demonstrated that the CFL and ATFL have a single confluent footprint on the anterior border of the distal fibula. Wenny et al. [1] also found that the fibular attachment of the CFL was suited direct adjacent to the fibular attachment of the ATFL. Therefore, these so-called anatomic reconstruction procedures could not fix the graft tendon at the original attachment point of ATFL and CFL anatomically. The reconstructed ligament in non-anatomic location will certainly have some effect on ankle rotational kinematics and kinetics during normal gait [23, 24]. In our study, we restored the ATFL and CFL anatomically from one common fibular origin, which better mimic the biological function of primary ligaments and should have resulted in more normal ankle kinematics. Besides, the graft in anatomic location is much likely to reduce soft tissue impingement and friction with lateral malleolus, articular surface of the talus, or peroneal tendon.

This procedure has several other advantages besides accurate anatomic localization. Creating a straight fibular tunnel is easier than previous ‘Γ’ shaped fibular tunnel [3, 22]. Furthermore, it spends less time with less intraoperative tunnel fracture probability. Two branch with a conjunct fibular outlet using Tightrope fixation will also reduce the risk of micromotion of the graft within the unfixed fibular tunnel, compared to previous bidirectional outlets that might have resulted in adjacent synovitis due to impingement or wearing of the graft. The traditional open techniques with larger incision put this anatomic region at a higher risk for colonization with microorganisms, nerve injury, ankle stiffness, and potential problems with wound healing than minimally invasive surgeries. This percutaneous technique has some merits for lateral ankle ligament reconstruction because it can access the same anatomic structures as an extensible approach without increasing the morbidity. Limited exposure reduces the likelihood of damage to the superficial peroneal nerve. The small incisions also have cosmetic appeal to young patients. It can be safely combined with arthroscopy as it preserves tissue structure, and allows early rehabilitation with less swelling and pain. Some argue that the real advantage of an open approach lies in simultaneous access to adjacent bones and tendons. Thus, in cases without these combined lesions, the open approach has no distinctive benefits. While compared to arthroscopic reconstruction, without complex threading and knotting, percutaneous method is easily reproducible, timesaving, and its learning curve is rapid. Meanwhile, it has the advantage of not preventing the use of arthroscopic methods, or open methods, in the case of failure.

Fixation using the Tightrope in our study is a relatively new technique compared with traditional methods. The Tightrope device was commonly used to offer cortical fixation for cruciate ligament reconstruction [25–27]. This suture-button system can facilitate folded graft fill of fibular tunnel and offer strong pullout strength. However, this system is not without its own problems. In our study, two patients reported soft tissue irritation from the cortical button. No granuloma formation or osteolysis in adjacent bone occurred in the patients.

This study had several limitations. First, the follow-up time was relatively short. The outcomes in this study are limited to the early results of treatment of CAI with a Tightrope system. We will continue to follow-up these patients. Second, the sample size was small. We are working on treating more patients with this procedure.

## Conclusion

Percutaneous anatomic reconstruction of the lateral ligaments of the ankle with a Tightrope system is an anatomic and effective procedure for the treatment of chronic lateral ankle instability. Good to excellent results can be obtained surgically with this procedure. It is a good option for CAI in a patient meeting our criteria of complex cases. Further studies are needed with a larger sample size and longer follow-up time.
